# Genome sequence of *Heterobasidion occidentale,* a fungus that causes annosus root and butt rot among conifer trees in North America

**DOI:** 10.1128/mra.00419-24

**Published:** 2024-08-23

**Authors:** Grace Sumampong, Nicolas Feau, Louis Bernier, Richard C. Hamelin, Jun-Jun Liu, Simon F. Shamoun

**Affiliations:** 1 Département des Sciences du bois et de la Forêt, Faculté de Foresterie et Géographie, Université Laval, Québec, Canada; 2 Natural Resources Canada, Canadian Forest Service, Pacific Forestry Centre, Victoria, British Columbia, Canada; 3 Department of Forest and Conservation Sciences, Faculty of Forestry, The University of British Columbia, Vancouver, British Columbia, Canada; University of California Riverside, Riverside, California, USA

**Keywords:** *Heterobasidion occidentale*, basidiomycete, fungal pathogen, conifer and broadleaf trees

## Abstract

We report an annotated draft genome of *Heterobasidion occidentale*, a fungus (Basidiomycota, Agaricomycetes) that has pathogenic and saprophytic lifestyles. This fungus belongs to the *H. annosum* (Fr.) Bref. sensu lato species complex that comprises several root rot pathogens. *Heterobasidion occidentale* causes annosus root and butt rot primarily in true fir (*Abies* spp.) and spruce (*Picea* spp.) species throughout western North America.

## ANNOUNCEMENT

*Heterobasidion annosum* (Fr.) Bref. sensu lato is a complex of five fungal species that cause annosus root rot in the Northern Hemisphere. The species native to North America, *H. irregulare* and *H. occidentale* (1), infect a wide host range, including both coniferous and broadleaf species (2). Their differentiation remains difficult because of overlapping morphological features, partial intersterility, and relies primarily on on-site ecology and host preference (1, 3). We used Illumina and Pacific Biosciences (PacBio) sequencing to generate a genome assembly for *H. occidentale* that will add to the three other reference genomes already sequenced in the *H. annosum* complex (4–7) and complement *H. occidentale* transcriptome data (8).

Pure culture of *H. occidentale* isolate DAOMC 252864 (Table 1) previously described as isolate B1436 (9) and PFC5313 (8) was grown in 2% malt extract broth (Difco) for 7–10 days at 20°C–22°C. Fresh mycelia (~50–100 mg) were rinsed, centrifuged, frozen in liquid nitrogen, and homogenized (FastPrep-24 5G; MP Biomedicals). DNA was extracted using a modified cetyltrimethylammonium bromide chloroform protocol with ethanol washes and isopropanol precipitation (10, 11) and quantified with Quant-iT PicoGreen dsDNA Assay Kit (Life Technologies), Tapestation, Bioanalyzer (Agilent), and Qubit (ThermoFisher). Identity was confirmed using *H. occidentale*-specific PCR oligonucleotide primers (3). The Illumina sequencing library was generated from 50 ng of quality-controlled DNA using the NEBNext Ultra II DNA Library Prep Kit (New England BioLabs). Libraries with desired insert size were quantified using the KAPA Library Quantification Kit (Kapa Biosystems) in a LabChip GX II (Perkin Elmer). The normalized pooled libraries (360 pM) were sequenced on a HiSeq 4000 for 2 × 100 cycles (paired-end mode) at the Génome Québec Innovation Center (Montréal, Canada). No shearing of DNA was performed before library preparation for PacBio Sequel Single-Molecule Real-Time (SMRT) sequencing technology (Pacific Biosciences) at the Génome Québec Innovation Center. SMRTbell DNA library (Pacific Biosciences) was constructed using 3.5 µg of DNA following manufacturer’s recommendations using the BluePippin Size-Selection System (Sage Science Inc.) with a cutoff range of 7–50 kb and cleaned up using AMPurebead before sequencing. *De novo* assembly of the 1.03 M PacBio reads using the haplotype-aware assemblers FALCON and FALCON-unzip v.0.3.0 (12) resulted in a pre-assembled genome with an N_50_ of 408,924 bp and a BUSCO completeness of 90.5%. The Illumina paired-end reads were then used to polish this pre-assembly with Pilon v.1.24 (13). Final ordering of contigs and scaffolding with RagTag v2.1 (14) resulted in the *de novo* assembly of 45.21 Mb distributed across 160 scaffolds with an N_50_ of 2.9 Mb (Table 1). Based on annotation using FunGAP Pipeline v1.1.0 (15) and RNAseq libraries from reference (8) (SRR6457920–SRR6457922), a total of 15,152 protein-coding genes (Table 1) were predicted in the final genome assembly of *H. occidentale* with an average CDS length of 415 bp (±356 bp). This assembly includes 780 potential secreted proteins, 285 putative carbohydrate-active enzymes (CAZymes), and 389 putative peptidases (Table 1). This genome assembly will further enhance our understanding of the pathogenicity of *H. occidentale* and interaction with its host.

**TABLE 1:** Origin, genome assembly, and annotation of *Heterobasidion occidentale*

Origin	
Host	*Abies concolor*
Location	Ouray County, Colorado, USA
DAOMC^*a*^ identifier	252864
Genome assembly^*b*^ and annotations	
Illumina reads	110.2 M
Illumina SRA	SRR28567167
PacBio reads	1.84 M
PacBio SRA	SRR28567168
PacBio-filtered reads	1.03 M
PacBio CCS reads	0.027 M
Total assembly size	45.21 Mb
GenBank assembly	JBCAQO010000000
Scaffolds	160
Largest scaffold	4,136,843 bp
N_50_	2,897,896 bp
GC %	52.8%
BUSCO completeness (%)	96.17%
Protein-coding genes	15,152
Secreted proteins	780^*c*^
CAZYmes	285^*d*^
Peptidases	389^*e*^

^*a*^ Canadian Collection of Fungal Cultures, Canadian Collection of Fungal Cultures (DAOMC) - agriculture.canada.ca.

^*b*^ Obtained by scaffolding the *H. occidentale* polished assembly against the *H. irregulare* genome GCA_000320585.2 (4) using RagTag v2.1 (14)*.*

^*c*^ Identified using SignalP5.0 and TargetP-2.0 (16, 17).

^*d*^ Identified using hmmer and Diamond against the dbCAN2 database (18).

^*e*^ Identified using hmmer and BLASTP search against the MEROPS database (19).

## Data Availability

All the data are available under BioProject number PRJNA1090464. The individual SRA and GenBank assembly numbers are included in Table 1. This Whole Genome Shotgun project has been deposited at DDBJ/ENA/GenBank under the accession JBCAQO000000000. The version described in this paper is JBCAQO010000000.
